# Genome Sequence of *Brevibacillus reuszeri* NRRL NRS-1206^T^, an l-*N*-Carbamoylase-Producing *Bacillus*-Like Bacterium

**DOI:** 10.1128/genomeA.01063-15

**Published:** 2015-09-17

**Authors:** Jie-ping Wang, Bo Liu, Guo-hong Liu, De-ju Chen, Ci-bin Ge, Zheng Chen, Jian-mei Che

**Affiliations:** Agricultural Bio-Resources Research Institute, Fujian Academy of Agricultural Sciences, Fuzhou, Fujian, China

## Abstract

*Brevibacillus reuszeri* NRRL NRS-1206^T^ is a Gram-positive, spore-forming, and strictly aerobic bacterium. Here, we report the draft 6.98-Mb genome sequence of *B. reuszeri* NRRL NRS-1206^T^, which is the first genome information of *B. reuszeri* and will provide useful information for genomic taxonomy and phylogenomics of *Bacillus*-like bacteria.

## GENOME ANNOUNCEMENT

The *Bacillus*-like strain NRRL NRS-1206^T^ was isolated by H. W. Reuszer and recorded initially as one strain of *Bacillus* brevi (now *Brevibacillus* brevi) (1). In 1995, the strain NRRL NRS-1206^T^ was identified as a unique species within the genus *Bacillus*, for which the name *Bacillus reuszeri* sp. nov. was proposed (1). In 1996, the genus *Brevibacillus* (within the family *Paenibacillaceae* but not the family *Bacillaceae*) was established by Shida et al.; meanwhile, *Bacillus reuszeri* was reclassified as *Brevibacillus reuszeri* comb. nov. (2). Up to now, very little research has been done on this spore-forming species. The only functional gene from *B. reuszeri* is l-methionine-*N*-carbamoylase, which will be a potential biocatalyst for the production of l-α-amino acids (3, 4). Given its taxonomic history and no available genomic information of *B. reuszeri*, its type strain NRRL NRS-1206^T^ was selected as one of the research objects in our genome sequencing project for genomic taxonomy and phylogenomics of *Bacillus*-like bacteria. Here, we present the high-quality draft genome sequence of *B. reuszeri* NRRL NRS-1206^T^ (DSM 9887).

To obtain the genome sequence of *B. reuszeri* NRRL NRS-1206^T^, two different DNA libraries with insert sizes of 500 and 5,000 bp were constructed and sequenced via the Illumina HiSeq 2500 system. After filtering the 1.15 Gb of raw data, the 1.00 Gb of clean sequence data were obtained, providing approximately 150-fold coverage. The reads were assembled via the SOAPdenovo software version 1.05 (5). Through the data assembly, 24 scaffolds consisting of 6,986,486 bp were obtained, and the scaffold *N*_50_ was 1,069,988 bp. The average length of the scaffolds was 291,103 bp, and the longest and shortest scaffolds were 2,212,757 bp and 536 bp, respectively. Moreover, 96.21% clean reads could be aligned back to the genome, by which 99.73% of the genome sequence could be covered.

The annotation of the genome was performed through the NCBI Prokaryotic Genomes Automatic Annotation Pipeline (PGAAP) using the GeneMark, Glimmer, and tRNAscan-SE tools (6). A total of 6,817 genes were predicted, including 6,463 coding sequences, 254 pseudogenes, 96 tRNAs, and 3 rRNA genes. There were 2,399 and 2,763 genes assigned to the COG and KEGG databases, respectively. The average DNA G+C content was 46.95%, which is in strong agreement with the value 46.5 mol% acquired by HPLC determination (1).

### Nucleotide sequence accession numbers.

This whole-genome shotgun project has been deposited at DDBJ/EMBL/GenBank under the accession number LGIQ00000000. The version described in this paper is the first version, LGIQ01000000.
